# Protective Mechanism of *Sea buckthorn* Proanthocyanidins Against Hydrogen Peroxide-Introduced Oxidative Damage in Adult Retinal Pigment Epithelial-19

**DOI:** 10.3390/antiox13111352

**Published:** 2024-11-05

**Authors:** Kaiyuan Ma, Michael Yuen, Tina Yuen, Hywel Yuen, Qiang Peng

**Affiliations:** 1 College of Food Science and Engineering, Northwest A&F University, Yangling, Xianyang 712100, China; makaiyuan@nwafu.edu.cn; 2 Puredia Limited, Xining 810000, Chinatina@puredia.com (T.Y.); hywel@puredia.com (H.Y.)

**Keywords:** retinal pigment epithelial, oxidative stress, *Sea buckthorn* proanthocyanidins, reactive oxygen species, mitochondrion

## Abstract

Retinal pigment epithelial (RPE) is an oxidation-resistant cell. But if it is subjected to various harmful stimuli for a prolonged period, an excessive amount of oxyradical will be generated to cause retinal dysfunction. We investigated and elucidated the protective mechanism of *Sea buckthorn* proanthocyanidins (SBP) against oxidative damage in RPE. In this study, we established an oxidative damage model of adult retinal pigment epithelial cell line-19 (ARPE-19) using hydrogen peroxide (H_2_O_2_), followed by different concentrations of SBP for 24 h. The finding demonstrated that SBP effectively inhibited the generation of malondialdehyde (MDA), restored the activity of superoxide dismutase (SOD) and content of glutathione (GSH), and significantly eliminated the level of reactive oxygen species (ROS) and oxidative stress. It was revealed that 100 µg/mL of SBP was more suitable for restoring oxidative damage in ARPE-19, which enhanced cell activity and migration ability and maintained normal cell morphology. In addition, SBP increased the expression of Bcl-2, decreased the expression of Bax and caspase-3, and activated the Nrf2/HO-1 signaling pathway to protect ARPE-19 from oxidative stress. Moreover, SBP could restore the morphology and quantity of mitochondria and inhibit mitochondrial permeability and swelling. The present results provide a theoretical basis for the protective and restorative effect of SBP in retinopathy caused by oxidative stress.

## 1. Introduction

Retinal pigment epithelium (RPE) is a highly differentiated monolayer pigment epithelial cell. It forms an important barrier to the eye along with structures such as choroid, Bruch’s membrane, and neural retina [1]. RPE has an essential effect in maintaining retinal integrity, establishing ocular immune function, and regulating early ocular growth [2]. In addition, it has major functions in retinaldehyde metabolism, phagocytosis, melanin synthesis and immunomodulatory effects [3]. RPE is an oxidation-resistant cell, but if it is subjected to various harmful stimuli such as environmental factors (bright light, smoking) and nutritional disorders for a prolonged period, it generates an excessive amount of oxyradical [4]. Once the amount of oxyradical exceeds the antioxidant capacity of RPE cells, a large amount of cumulative oxidative damage occurs, causing apoptosis of cells. RPE cells suffer irreparable damage to their nucleic acids, phospholipids, proteins, and organelles, such as mitochondria, causing retinal dysfunction and resulting in retinopathy of prematurity (ROP) and diabetic retinopathy (DR), as well as age-related macular degeneration (AMD) [5,6,7].

The formation of oxidative stress is related to reactive oxygen species (ROS) and concurrently impacts the content and activity of antioxidants and oxidative enzymes in cells [8]. Yildirim et al. [9] demonstrated that AMD reduced antioxidant defenses and increased oxidative stress in vivo by measuring the activities of superoxide dismutase (SOD) and glutathione peroxidase (GSH-Px), as well as oxidized protein products, malondialdehyde (MDA), glutathione (GSH), and vitamin C in 25 patients with AMD. In addition, mitochondria are the organelles in which cells produce the most ROS. Mitochondria activates caspases by releasing apoptotic zymogen and other activating factors. This pathway mainly involves the formation of the Bax/Bcl-2 complex, which affects the permeability of the mitochondrial membrane and alters the surface potential. Significant alteration of mitochondrial osmotic pressure results in the release of a large amount of cytochrome C, leading to the death or apoptosis of RPE [10]. Mitochondrial dysfunction driven by oxidative stress is a critical driver of retinal diseases.

The isolation and extraction of natural active ingredients and their therapeutic and health effects have been a hot topic. In recent years, more and more researchers have indicated that plant extracts exhibit protective behavior on retinal cells. *Sea buckthorn* (*Hippophae rhamnoides* Linn.) contains more than 190 active ingredients and nutrients, including vitamins, flavonoids, polyphenols, terpene, proanthocyanidin, etc. It has been widely used in the field of food and in the health field [11]. Currently, proanthocyanidins are used for a variety of diseases due to their various pharmacological activities, such as hypoglycemic [12], anticancer [13], and cardiovascular disease prevention [14]. According to Chang et al., proanthocyanidins in cranberries could enhance cell vitality and repair ability and have a good free radical scavenging activity in the model of AMD on ARPE-19 cells [15]. Wang et al. [16] proved that proanthocyanidins of *Lotus seedpod* possessed favorable anti-apoptosis, neuroprotective effects and antioxidative stress, which could heavily alleviate the retinal damage caused by light. A large amount of literature indicated that the stems, leaves, flowers, roots, seeds and fruits of *Sea buckthorn* had high concentrations of proanthocyanidins [17]. *Sea buckthorn* proanthocyanidins (SBP) have strong antioxidant effects and cardiovascular protection, anti-aging, and anti-ulcer effects. Widen et al. [18] found that *Sea buckthorn* had better protective effects on erythrocytes from oxidative stress than other berries. Therefore, the purpose of our research was to explore and describe the protective mechanism of SBP on oxidative-damaged RPE cells.

RPE cells produce large amounts of H_2_O_2_ when performing daily functions. As a metabolite in vivo and as a ROS, it is commonly utilized to induce oxidative stress and apoptosis due to its trait to easily induce free radical production through the cell membrane and lipid peroxidation [19]. In this experiment, we used ARPE-19 cells and H_2_O_2_ to establish an oxidative-damaged model. SBP was added to protect the cells from oxidative damage. The protective effect and mechanism of SBP against H_2_O_2_-introduced oxidative damage in APRE-19 cells were elucidated from multiple aspects. The study offers a new perspective on the cure of retinopathy.

## 2. Materials and Methods

### 2.1. Materials

*Sea Buckthorn* proanthocyanidins (SBP, Purity: 91.5%; Trademark: CyanthOx^TM^) were purchased from Puredia Limited (Qinghai, China). Adult retinal pigment epithelial cell line-19 (ARPE-19, ATCC, IM-H264), ARPE-19 specific culture medium (IM-H264-1), PBS buffer fluid (IMC-401) and 0.25% trypsin digestive fluid does not contain EDTA and phenol red (IMC-506-100 mL) were provided by Xiamen Immocell Biotechnology Co., Ltd. (Xiamen, Fujian Province, China). Cell Counting Kit 8 (CCK-8) cell proliferation and cytotoxicity assay kit (CA1210) and reactive oxygen species (ROS) assay kit (CA1410) were obtained from Beijing Solarbio Science & Technology Co., Ltd. (Beijing, China). SOD assay kit, GSH assay kit, MDA assay kit, Nitric oxide (NO) detection kit (S0021S), Mito-Tracker Red CMXRos (C1035), and mitochondrial permeability transporter pore (mPTP) detection kit (C2009S) were obtained from Beyotime Biotech Inc. (Shanghai, China). Senescence β-Galactosidase Staining Kit (C0602) was purchased from MultiSciences Biotech Co., Ltd. (Hangzhou, Zhejiang Province, China).

### 2.2. ARPE-19 Cell Culture

ARPE-19 cells were cultured with ARPE-19 specific culture medium (D/F12 + 10% FBS + PS) at 37 °C with 5% CO_2_. Passaging culture was performed when cell confluency reached 80–90%. After that, the supernatant was separated from the medium, and the cells were washed 1–2 times using PBS buffer. Next, 1 mL of digestion solution was added for infiltrating all cells. The digestion was terminated when the cells became rounded and detached. Finally, the cells were centrifuged at 1000 rpm for 5 min, the supernatant was separated once more, and the cell suspension was subcultured at a 1:2 volume ratio.

### 2.3. Cell Viability

Cell viability was assessed by CCK-8 assay. The drugs were introduced to the 96-well culture plates (100 μL per well) containing cells and allowed to incubate for 24 h. Following this, 10 µL of CCK-8 solution was pipetted into every plate well. By continuing a further 2 h incubation, the absorbance was recorded using a microplate reader (PowerWave XS, Bio-Tek, Winooski, VT, USA) at 450 nm.

### 2.4. H_2_O_2_-Induced Oxidative-Damaged Model

ARPE-19 cells under the log growth phase were inoculated into 96-well plates with 100 μL per well at a concentration of 1.0 × 10^5^ cells/mL. When the cells were adhered after 24 h incubation, the supernatant was removed, and H_2_O_2_ diluted in ARPE-19 cell-specific culture medium was added to different concentrations of 100, 200, 300, 400, 500, 600, 700, 800 µmol/L, respectively. Finally, the cell viability was measured by CCK-8 assay to identify the precise H_2_O_2_ concentration after culturing for 24 h.

(1) $$\mathrm{Cell}\;\mathrm{viability}\;(\%)=\frac{{\mathrm{OD}}_{\mathrm{sample}}-{\mathrm{OD}}_{\mathrm{blank}}}{{\mathrm{OD}}_{\mathrm{control}}-{\mathrm{OD}}_{\mathrm{blank}}}\times 100\%$$

### 2.5. Cytotoxicity of SBP

ARPE-19 cells under the log growth phase were inoculated into 96-well plates with 100 μL per well at a concentration of 1.0 × 10^5^ cells/mL. When the cells were adhered after 24 h incubation, the supernatant was removed, and the cells were rinsed twice by PBS. Subsequently, 100 µL of SBP prepared in ARPE-19-specific culture medium were added at concentrations of 50, 100, 200, 400, 800, and 1200 µg/mL. Cytotoxicity of SBP, i.e., cell viability, was also measured with CCK-8 assay.

### 2.6. Cell Treatment

ARPE-19 cells under the log growth phase were inoculated into 96-well plates with 100 μL per well at a concentration of 1.0 × 10^5^ cells/mL. When the cells were adhered after 24 h incubation, the supernatant was removed, the cells were rinsed twice using PBS, and they were treated with drugs. There were 5 test groups: (1) Control group: only ARPE-19 specific culture medium; (2) Oxidative-damaged model group: ARPE-19 culture medium including 600 µmol/L of H_2_O_2_; (3) ARPE-19 culture medium including 600 µmol/L of H_2_O_2_ and 25 µg/mL of SBP; (4) ARPE-19 culture medium including 600 µmol/L of H_2_O_2_ and 50 µg/mL of SBP; (5) ARPE-19 culture medium including 600 µmol/L of H_2_O_2_ and 100 µg/mL of SBP. After 24 h of incubation, cell morphology was obtained by an inverted microscope, and then the cell viability was measured by the CCK-8 assay.

### 2.7. Reactive Oxygen Species Level

The above five test groups were detected by ROS assay kit for ROS assay. Specific operation: the supernatant was separated and rinsed twice with PBS. ARPE-19-specific culture medium (with 10 µM of DCFH) without serum was added to culture plates. After 30 min of cultivation, the cells were rinsed 3 times with PBS, then visualized with a green fluorescence microscope (IX71-F22FL/PH, Olympus, Tokyo, Japan). Then, the cells of every plate well were pipetted and measured by an F-7000 fluorescence spectrophotometer (Hitachi, Tokyo, Japan) with an emission wavelength of 530 nm and an excitation wavelength of 485 nm.

### 2.8. SOD Activity, GSH, and MDA Content

The supernatant was removed after the culture of the above five test groups. Next, 200 µL cell lysate was pipetted into the culture plate, then gathered and centrifuged for 10 min at 12,000 rpm and 4 °C. The supernatant was pipetted as the sample to be tested. Finally, SOD activity and GSH MDA contents were determined according to the specific experimental procedure described in the kit.

### 2.9. Cell Wound Scratch

Digested ARPE-19 cells were inoculated into 6-well plates at a concentration of 1.0 × 10^5^ cells/well until cells adhered. When the number of cells was sufficient to cover the entire bottom of the plate, a 10 µL pipette tip perpendicular to the culture plate was used to create cell wound scratches, trying to ensure that every wound scratch was the same width. Subsequently, the supernatant was separated, and ARPE-19 cells were rinsed 3 times using PBS to clean cell debris from the wound scratches. Next, the culture medium without serum containing 600 µmol/L H_2_O_2_ or 100 µg/mL of SBP was introduced for three treatment groups: control, H_2_O_2_, and H_2_O_2_-SBP. Cell migration was visualized by an inverted microscope at ×20 magnification after culturing for 24 h, and the cell migration rate was calculated using the following equation [20]:

(2) $$\mathrm{Cell}\;\mathrm{migration}\;\mathrm{rate}\;(\%)=\frac{0\;h\;\mathrm{scratch}\;\mathrm{width}-\mathrm{scratch}\;\mathrm{width}\;\mathrm{after}\;\mathrm{cultivation}}{0\;h\;\mathrm{scratch}\;\mathrm{width}}\times 100\%$$

### 2.10. Western Blot

The proteins of control, H_2_O_2_, and H_2_O_2_-SBP treatment groups were isolated by gel electrophoresis and then transferred to PVDF membrane. They were blocked for 2 h, rinsed 5 times using TBST, and incubated with primary antibody overnight. Subsequently, they were incubated with secondary antibody for 2 h and rinsed 5 times using TBST again. Protein bands were obtained by the ECL Western Blot Detection Kit (1:5000, Gsebio, Xi’an, Shanxi, China) [21].

### 2.11. Mitochondrial Assay

#### 2.11.1. Micromorphology

After culture treatment, ARPE-19 cells were immobilized by 2.5% glutaraldehyde for 2 h, followed by rinsing 3 times with 0.1 M PBS, then immobilized by 1% osmium tetroxide for 1 h. Later, gradient ethanol was used for dehydration of the cells, which were immersed in epoxy resin overnight at 37 °C and polymerized at 60 °C for 48 h. The slices were then dyed with uranyl acetate and put into distilled water by stirring to wash thoroughly. Then, the slices were float-dyed with lead citrate and washed with distilled water. They were air-dried on filter paper, and micromorphologies were observed by transmission electron microscope (JEM-1230, Jeol, Tokyo, Japan).

#### 2.11.2. Quantity

The number of mitochondria of ARPE-19 cells was tested by mitochondrial red fluorescent probe Mito-Tracker Red CMXRos. After discarding the culture fluid, Mito-Tracker Red CMXRos reagent was added and cultured for 20 min at 37 °C and replaced with fresh cell culture medium pre-warmed at 37 °C. Finally, a fluorescence microscope was used for observation, and the mean fluorescence intensity was determined by Image J software (https://imagej.net/ij/, Fiji, NIH, Bethesda, MD, USA).

#### 2.11.3. Permeability

ARPE-19 cells were inoculated into cell culture plates for an mPTP detection kit until the cells grew to reach an appropriate confluency for drug treatment. Then, the culture fluid was removed, and ARPE-19 cells were rinsed twice using PBS. Fluorescence quenching working solution and Calcein AM staining solution were used so that all cells were evenly covered. After culturing for 30 min at 37 °C, the used culture medium was exchanged for fresh culture medium pre-warmed at 37 °C and incubated for a further 30 min until Calcein AM was completely hydrolyzed by intracellular esterases to generate green, fluorescent Calcein. The culture fluid was removed, and the cells were rinsed 3 times using PBS and added to the assay buffer. The fluorescence microscope was used to observe the permeability of mitochondria, and the mean fluorescence intensity was determined by Image J software (Fiji, NIH, Bethesda, MD, USA).

#### 2.11.4. Swelling Level

Isolation of mitochondria: (1) Cells were seeded on cell culture plates and drug-treated until cells grew to reach a appropriate confluency for drug treatment; (2) The culture fluid was separated and cells were rinsed 3 times using ice-cold PBS; (3) After adding 1 mL of fresh ice-cold PBS, the adherent cells were scraped into a fresh 1.5 mL centrifuge tube and centrifuged for 10 min at 800 rpm and 4 °C; (4) The supernatant was separated, the precipitate was obtained by adding 1 mL mitochondrial isolation buffer and incubating for 30 min on ice; (5) The cells were homogenized manually up and down on ice water with a glass homogenizer; (6) The homogenate was pipetted to a 1.5 mL centrifuge pipe and centrifuged 3 times for 5 min at 1000 rpm and 4 °C; (7) The supernatant was discarded, the precipitate with mitochondria was resuspended using 1 mL of mitochondrial isolation buffer; (8) The mitochondria were centrifuged at 14,000 rpm and 4 °C for 15 min; (9) The concentration of mitochondrial proteins was measured by the BCA method.

Assay of Ca^2+^-induced mitochondrial swelling: (1) To induce the swelling of mitochondria, 10 μL of 20 mM CaCl_2_ aqueous solution containing 500 nmol/mg mitochondrial protein was added; (2) The absorbance of different treatment groups was determined by a microplate reader at 540 nm.

### 2.12. Statistical Analysis

Each trial was replicated 3 times, and the results were expressed as means and standard deviations. Statistical comparisons were used with the Tukey post hoc test and one-way analysis of variance (ANOVA) after the normal distribution test. Different letters indicate significant differences at *p* < 0.05 among groups. All data were carried out using SPSS 22.0 (SPSS Inc., Chicago, IL, USA).

## 3. Results

### 3.1. Establishment of H_2_O_2_-Induced Oxidative Damage Model and Cytotoxicity of ARPE-19 Treated by SBP

**Figure 1:**
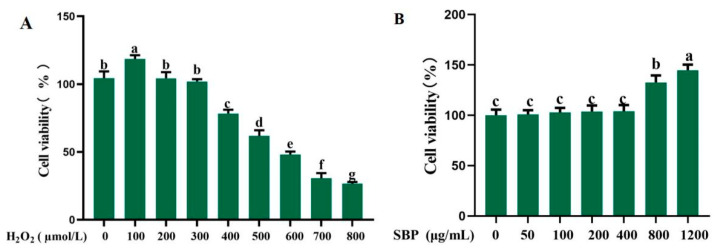
Establishment of H_2_O_2_-induced oxidative-damaged model and cytotoxicity of ARPE-19 cells treated by SBP. (**A**) Cell viability of ARPE-19 treated by different concentrations of H_2_O_2_; (**B**) Cytotoxicity of ARPE-19 treated by different concentrations of SBP. Different letters indicate significant difference (*p* < 0.05).

The cell viability of ARPE-19 cells treated by H_2_O_2_ is illustrated in Figure 1A. H_2_O_2_ increased cell viability at a low concentration of 100 µmol/L, while cell viability was significantly reduced from the concentration of H_2_O_2_ higher than 400 µmol/L. The linear equation between H_2_O_2_ concentration and cell viability was Y = 0.1494X + 136.68. According to the equation, the IC_50_ value is 580.2 µmol/L. As a result, the concentration of 600 µmol/L H_2_O_2_ was selected to establish an oxidative-damaged model in the subsequent experiments.

Figure 1B shows the cytotoxicity of ARPE-19 cells treated by SBP. SBP was non-toxic in the concentration range of 0–1200 µg/mL. The concentration of SBP below 400 µg/mL did not make a significant difference in cell viability, but SBP concentration above 800 µg/mL promoted the proliferation of ARPE-19. To ensure consistency in cell numbers among treatment groups, it is reasonable to choose a concentration of SBP below 400 µg/mL.

### 3.2. Cell Viability and Cell Morphology

**Figure 2:**
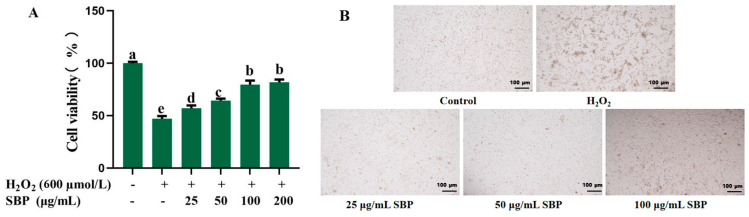
The cell viability (**A**) and morphology (**B**) of H_2_O_2_-induced oxidative-damaged cells treated by different concentrations of SBP. In the figure, “+” means added, “-” means not added. Different letters indicate significant difference (*p* < 0.05).

The cell viability of oxidative-damaged cells treated with different concentrations of SBP is presented in Figure 2A. Cell viability was significantly decreased by H_2_O_2_ compared with the control group. SBP gradually increased the cell viability with the increase of SBP concentration at 25 µg/mL to 100 µg/mL, but there was no obvious difference in cell viability when the concentration of SBP exceeded 100 µg/mL. Therefore, SBP concentrations between 25–100 µg/mL are suitable to detect relevant indexes.

As seen in Figure 2B, significant alteration in cell morphology is observed in the H_2_O_2_-induced oxidative-damaged model compared to the control; the intercellular gap was enlarged and wrinkled, and some of the cells died. With the increasing of SBP concentrations, the cell gap was reduced, the cell boundary was more rounded, the number of viable cells obviously increased, and the number of dead cells was reduced. The results of morphology indicated that SBP protected ARPE-19 cells from oxidative stress-induced damage by inhibiting cell death and preserving morphology.

### 3.3. ROS, SOD Activity, GSH, and MDA Content

**Figure 3:**
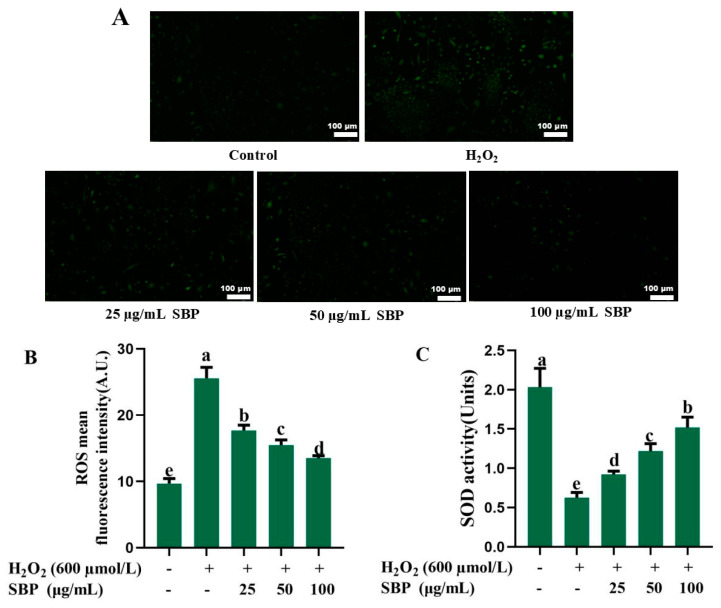

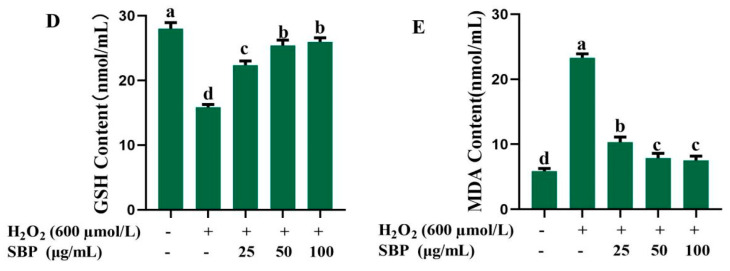
ROS level, SOD activity, GSH and MDA content of H_2_O_2_-induced oxidative-damaged cells treated by SBP. (**A**) ROS detection fluorescence images of ARPE-19 cells under different experimental conditions; (**B**–**E**) ROS level, SOD activity, GSH, MDA content of ARPE-19 cells under different experimental conditions. In the figure, “+” means added, “-” means not added. Different letters indicate significant difference (*p* < 0.05).

We measured the ROS level, SOD activity, GSH, and MDA content of ARPE-19 cells to explore the antioxidant effect of SBP in the oxidative damage model. Fluorescence images and ROS levels of ARPE-19 cells are exhibited in Figure 3A,B. Compared with the control group, the H_2_O_2_-induced oxidative-damaged model showed an obvious increase in fluorescence intensity, proving the existence of plenty of ROS. The fluorescence intensity of the SBP-treated group was lower than the H_2_O_2_ group, and the ROS level tended to decrease with the increase in SBP concentration. Figure 3C–E illustrated that SOD activity and GSH content of the H_2_O_2_-introduced injured group was much reduced, and MDA content was much improved compared to control. But with the increasing of SBP concentrations, SOD activity and GSH content of ARPE-19 treated with SBP improved, and MDA content reduced gradually.

The above results indicated that SBP-treated groups could effectively restore SOD activity and GSH content, inhibit the generation of MDA, effectively eliminate ROS, reduce oxidative stress, contribute to maintaining normal cell morphology, and protect ARPE-19 cells. The SBP concentration of 100 µg/mL was decided for subsequent experiments.

### 3.4. Migration Ability

**Figure 4:**
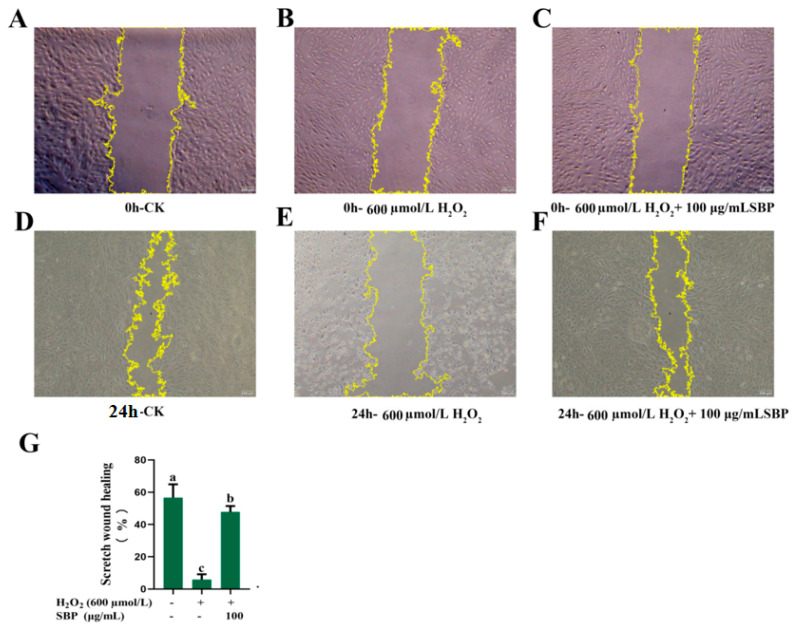
Migration ability of H_2_O_2_-induced oxidative-damaged cells treated by SBP (magnification, ×20). (**A**) Micrograph of control group at 0 h of cultivation; (**B**) Micrograph of ARPE-19 cells treated by 600 µmoL/L H_2_O_2_ at 0 h of cultivation; (**C**) Micrograph of ARPE-19 cells treated by 600 µmoL/L H_2_O_2_ and 100 µg/mL SBPat 0 h of cultivation; (**D**) Micrograph of control group after 24 h of cultivation; (**E**) Micrograph of ARPE-19 cells treated by 600 µmoL/L H_2_O_2_ after 24 h of cultivation; (**F**) Micrograph of ARPE-19 cells treated by 600 µmoL/L H_2_O_2_ and 100 µg/mL SBP after 24 h of cultivation; (**G**) Cell migration rate of the above three treatment groups after 24 h of cultivation. In the figure, “+” means added, “-” means not added. Different letters indicate significant difference (*p* < 0.05).

To a certain extent, the cell wound scratch simulates the migration process of cells in vivo. By observing whether the surrounding cells grow or repair to the central area of wound scratch, the growth, repair, and migration ability of cells can be judged. Figure 4A–G represents the micrographs of cell migration by microscopy and the quantified cell migration rates, respectively. After 24 h of incubation, the migration rate of the oxidative-damaged model was considerably smaller than that of the control group. However, the migration rate of the oxidatively damaged model was greatly increased when adding SBP, which was close to that of the blank group. SBP possesses a good ability to promote the migration and repair abilities of ARPE-19 cells.

### 3.5. Antioxidant-Related Signaling Pathways by Western Blot

#### 3.5.1. Apoptosis-Related Proteins

**Figure 5:**
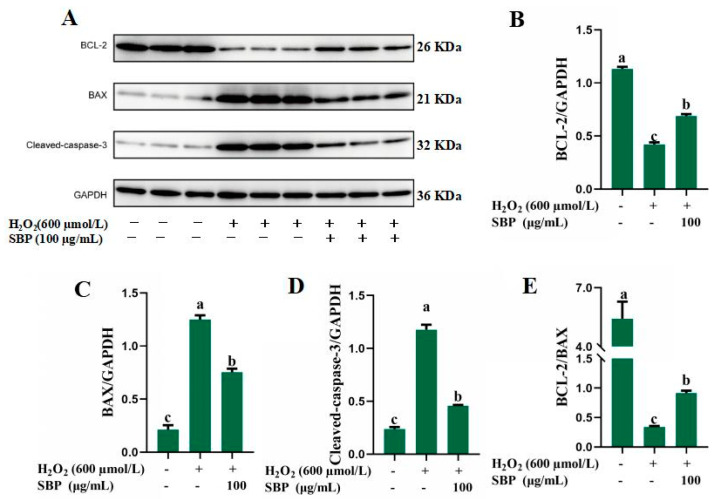
Apoptosis-related proteins of H_2_O_2_-induced oxidative-damaged cells treated by SBP. (**A**) Protein bands of related proteins by Western blot; (**B**–**D**) The expression of Bcl-2, Bax, and caspase-3, respectively; (**E**) The expression of Bcl-2/Bax heterodimerin. In the figure, “+” means added, “-” means not added. Different letters indicate significant difference (*p* < 0.05).

In this study, three apoptosis-related proteins, Bcl-2, Bax, and caspase-3, were selected and observed. Figure 5A–E reveals the protein bands and expression of Bcl-2, Bax, caspase-3, and Bcl-2/Bax, respectively. This section demonstrated that compared with the control group, Bax and caspase-3 expression of ARPE-19 treated by H_2_O_2_ effectively increased, while Bcl-2 expression was significantly reduced. After introducing 100 µg/mL SBP to the oxidative-damaged model, Bax and caspase-3 expression were decreased, and Bcl-2 expression was significantly increased. Figure 5E shows that SBP significantly improves the ratio of Bcl-2 and Bac proteins in the mitochondria and generates more stable Bcl-2/Bax heterodimers. These results demonstrate that SBP protects retinal cells through the mitochondrial pathway.

#### 3.5.2. Nrf2/HO-1 Pathway

**Figure 6:**
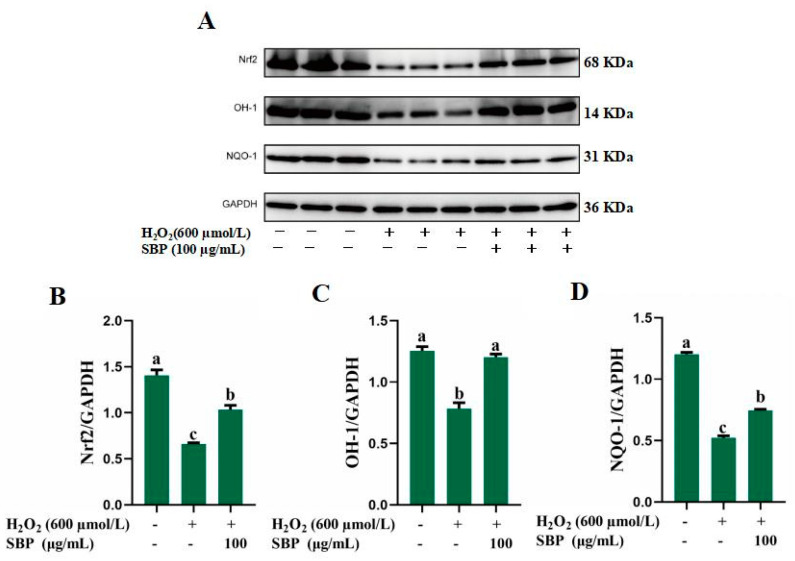
Nrf2/HO-1 pathway of H_2_O_2_-induced oxidative-damaged cells treated by SBP. (**A**) Protein bands of Nrf2/HO-1 pathway-related proteins; (**B**–**D**) The expression of Nrf2, HO-1, and NQO-1, respectively. In the figure, “+” means added, “-“ means not added. Different letters indicate significant difference (*p* < 0.05).

Nrf2/HO-1 is a major antioxidative stress pathway of the human body, and activation of this pathway has a protective effect on oxidatively damaged cells. Figure 6 shows the protein bands and expression of Nrf2/HO-1 pathway-related proteins. The results indicated that the expression of nuclear factor erythroid 2-related factor 2 (Nrf2), heme oxygenase-1 (HO-1), and NAD(P)H quinone oxidoreductase-1 (NQO-1) at the protein level was significantly reduced after H_2_O_2_ treatment, which implied the inhibiting of Nrf2/HO-1 pathway. After the addition of SBP in H_2_O_2_-induced damaged cells, the expression of pathway-related proteins was substantially increased. This suggests that SBP restores the cell from H_2_O_2_-induced oxidative damage to a normal redox state.

### 3.6. Cellular Mitochondria

#### 3.6.1. Mitochondrial Micromorphology

**Figure 7:**
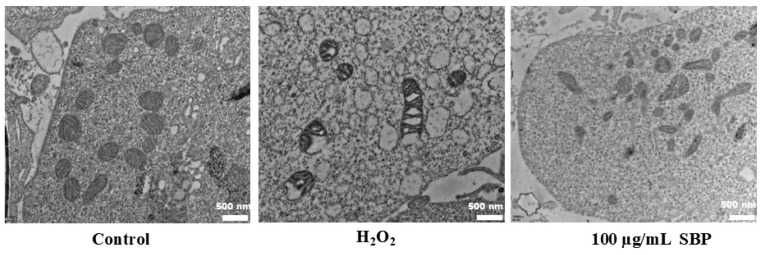
Mitochondria micromorphology of H_2_O_2_-induced oxidative-damaged cells treated by SBP (21,000×).

As shown in Figure 7, normal mitochondria were tubular or spherical with a regular arrangement of internal cristae, whereas mitochondria in H_2_O_2_-induced oxidative-damaged cells were injured with mitochondrial swelling and the internal vacuoles appearing inside. In the SBP treatment group, although some of the mitochondrial cristae were blurred, no vacuoles appeared, indicating that SBP had a good protective effect.

#### 3.6.2. Mitochondrial Quantity

**Figure 8:**
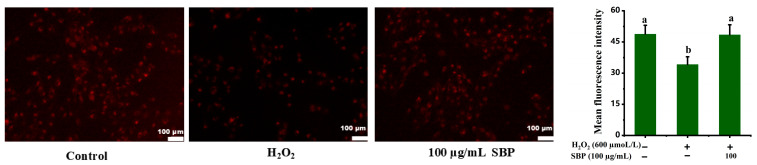
Mitochondria quantity of H_2_O_2_-induced oxidative-damaged cells treated by SBP. Different letters indicate significant difference (*p* < 0.05).

Excessive hydrogen peroxide contributes to the mitochondrial metabolic burden, causing high production of ROS and inhibiting mitochondrial biosynthesis [10]. Figure 8 shows the mitochondrial quantity of H_2_O_2_-induced oxidative-damaged cells treated by SBP. It was seen that compared with the control group, H_2_O_2_-induced oxidative-damaged cells significantly reduced the fluorescence intensity and decreased mitochondrial quantity. When SBP was introduced to the oxidative-damaged model, the red fluorescence intensity was significantly increased, and the mitochondrial quantity was effectively restored. As such, it was concluded that oxidative damage of mitochondria was inhibited.

#### 3.6.3. Mitochondrial Permeability

**Figure 9:**
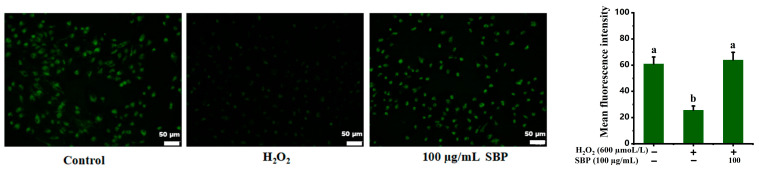
Mitochondria permeability of H_2_O_2_-induced oxidative-damaged cells treated by SBP. Different letters indicate significant difference (*p* < 0.05).

As can be seen in Figure 9, the green fluorescence intensity of the oxidative-damaged group decreased compared with the control group. Oxidative stress induced by H_2_O_2_ treatment caused the disruption of the mitochondrial membrane structure and the absence of mitochondrial membrane potential, causing the opening of mPTP, leading to the swelling, dysfunction, and metabolic imbalance of mitochondria. Nevertheless, the fluorescence intensity was restored after adding SBP to the oxidative-damaged model, demonstrating that SBP can improve mitochondrial permeability by preventing the opening of mPTP.

#### 3.6.4. Mitochondrial Swelling Degree

**Figure 10:**
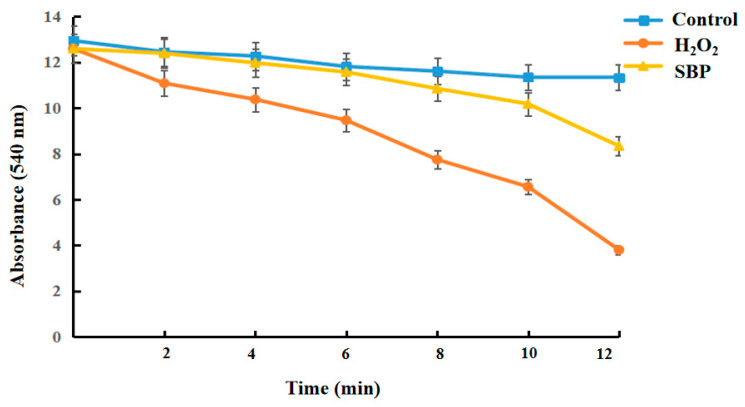
Mitochondria swelling of H_2_O_2_-induced oxidative-damaged cells treated by SBP.

In the mitochondria-Ca^2+^ system, the faster the rate of decrease in absorbance, the deeper the swelling of mitochondria and the more serious the impairment of mitochondrial function. Figure 10 proved that the absorbance of the H_2_O_2_-introduced damaged model decreased faster than that of the control group, leading to mitochondrial swelling. In contrast, the addition of SBP slowed down the rate of decrease and effectively inhibited mitochondrial swelling.

## 4. Discussion

**Figure 11:**
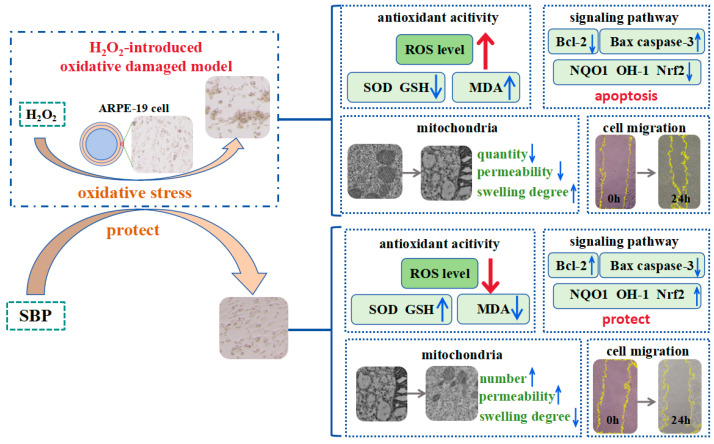
The protective mechanism of SBP on H_2_O_2_-introduced oxidative-damaged RPE model.

RPE has many important roles in the eye. It can not only regulate retinal development but also reduce photo-oxidative stress by absorbing excessive scattered light as it enters the eye. Meanwhile, RPE facilitates the secretion of growth factors, regulates the immune response of the eye, and transports metabolic wastes through Burch’s membrane to the choroid for elimination [22]. However, if RPE is subjected to harmful stimuli from the external environment for a long period of time, oxidative damage will be caused, resulting in apoptosis of RPE cells, retinal dysfunction, and various retinal diseases [23]. During the process of oxidative stress, the main region of ROS generation is the mitochondria, and oxidative stress can contribute to the death of RPE by causing damage to mitochondria. Therefore, how to combat oxidative damage and protect RPE cells and mitochondrial function has become one of the important tasks in retina research. SBP has favorable antioxidant properties and physiological activities; it is utilized in a variety of applications, such as food and medicine. Our team previously found that SBP significantly protected RAW264.7 cells from oxidative stress introduced by H_2_O_2_ [24]; however, the detailed protective mechanism required further study. We used ARPE-19 cells and 600 µmol/L H_2_O_2_ to develop an oxidatively damaged model, and SBP treatment groups were prepared by treating ARPE-19 cells using 25, 50, and 100 µg/mL of SBP. Figure 2 and Figure 4 indicate that SBP effectively restores cell morphology and improves cell viability and migration. We aimed to study the protective mechanism of SBP in H_2_O_2_-induced oxidative damage in ARPE-19 cells (Figure 11).

Excessive generation of ROS may damage lipids, proteins, and DNA. Research has found that SBP can increase the activity of antioxidant enzymes and utilize multiple non-enzymatic defense mechanisms to remove excessive ROS produced by H_2_O_2_-induced oxidative stress, thereby inhibiting oxidative damage [25]. Antioxidant enzymes primarily consist of glutathione (GSH), superoxide dismutase (SOD), etc., which perform a vital function in the balance of oxidation and antioxidation in vivo. SOD can defend the cells from damage by eliminating excessive free radicals [26]. GSH, which is also a significant free radical scavenger, is important in detoxification, antioxidant, antitumor activity and enhancement of immunity [27]. MDA, as a kind of lipid peroxide, can indirectly reveal the level of cellular damage by displaying the lipid peroxidation level [28]. Therefore, the activity of SOD and the content of GSH and MDA can reflect the antioxidant capacity of the cells. In our research, SOD activity and GSH content of the H_2_O_2_ treated group were lower, and MDA content was higher than that of the control group, which led to a significant increase in ROS level. After adding SBP at different concentrations, it was impressive to discover that SBP gradually improved SOD activity and GSH content and inhibited the production of MDA. SBP reduced the stimulatory responses of oxidative stress by removing excessive ROS, resulting in protecting ARPE-19 from oxidative damage.

The mitochondrial apoptosis pathway is activated in our model. In the oxidatively damaged model, the pro-apoptotic protein of Bcl-2 on the mitochondrial membrane surface is activated, which leads to changes in mitochondrial permeability, causing the release of cytochrome C into the cytoplasm and apoptosis [29]. This experiment selected Bcl-2, Bax and caspase-3 as three key apoptosis-related proteins on the mitochondrial apoptosis pathway. The upregulation of Bax expression suggests that cells are in the apoptotic phase, while Bcl-2 and Caspase-3 can inhibit apoptosis caused by multiple cytotoxic factors, and their expression can enhance the resistance to most cytotoxic factors [30]. In our study, we observed Bax and caspase-3 expression enhanced significantly in the injury group, while Bcl-2 expression reduced. After the introduction of SBP, Bcl-2 expression enhanced significantly, and Bax and caspase-3 expression reduced. These results indicate that H_2_O_2_ induced mitochondrial apoptotic pathway in ARPE-19 cells, but SBP inhibited this mitochondrial pathway. Meanwhile, SBP improved the ratio of Bcl-2 and Bax on the mitochondrial membrane surface, and Bax/Bax dimer dissociated in large amounts, generating a more stable Bcl-2/Bax heterodimer, which counteracted its apoptosis-inducing effect and prolonged the cell survival period [31].

In addition, as a vital antioxidative stress pathway, activation of the Nrf2/HO-1 signaling pathway has a protective effect on oxidative damage. Nrf2, which is a crucial transcription factor in Nrf2/HO-1 signaling pathway, assists in regulating the expression of phase II antioxidant enzymes, including SOD, HO-1, and NQO-1. These enzymes can process or remove carcinogens or toxins and regulate the transcription of a number of genes with direct or indirect antioxidant effects [32]. NQO-1 and HO-1 can degrade heme and quinone, which are direct sources of free radicals and have the ability to transfer electrons [33]. Figure 6 shows that the Nrf2/HO-1 signaling pathway was inhibited by H_2_O_2_ treatment, while Nrf2, HO-1 and NQO-1 expression at the protein level was significantly improved in the SBP treatment group. SBP enhanced the expression of multiple antioxidant-responsive enzymes and restored the cells from H_2_O_2_-induced oxidative stress to a normal redox state. It suggests that the protective effect of SBP against oxidative stress in ARPE-19 cells may be achieved by activating the Nrf2/HO-1 pathway.

Approximately 90% of ROS in the cell are originated from the mitochondrial respiratory chain. Oxidative stress can cause changes in mitochondrial structure, change the potential of the mitochondrial membrane and open the mPTP, which results in increased permeability of the mitochondrial membrane and mitochondrial swelling and dysfunction [34]. mPTP is an essential structure in the mitochondrial membrane, and its extent of opening is critical to cellular metabolism [35]. Excessive opening of mPTP not only leads to mitochondrial swelling and a decrease in mitochondrial membrane potential but also impairs the normal activity of the mitochondrial respiratory chain, decreases the production of ATP, and increases the production and release of ROS [36]. This research about mitochondria demonstrated the opening extent of mPTP increased when ARPE-19 cells were treated with H_2_O_2_, mitochondrial membrane potential decreased, and oxidative damage led to swelling of mitochondria, resulting in mitochondrial dysfunction and metabolic imbalance. In contrast, SBP significantly inhibited mitochondrial oxidative damage, restored the degree of mPTP opening and the number and morphology of mitochondria, inhibited mitochondrial swelling, protected mitochondrial function and enhanced cell viability. Thus, we have demonstrated in multiple ways that SBP can essentially restore the damage of ARPE-19 due to oxidative stress and illustrate the involved mechanisms. In the follow-up experiment, SBP will be tested further in animal models of RPE damage or in clinical trials of AMD to visualize the protective effect of SBP.

## 5. Conclusions

The study simulated the oxidative damage of ARPE-19 with H_2_O_2_ and clarified the protective mechanism of SBP against H_2_O_2_-introduced oxidative damage in ARPE-19 cells. The results showed that SBP could effectively scavenge excess ROS, reduce intracellular oxidative stress, improve cell viability, and restore cell morphology and migration. Additionally, 100 µg/mL of SBP was efficient for the repair of oxidative damage in retinal cells. We found that SBP increased the expression of anti-apoptotic protein Bcl-2, decreased the expression of pro-apoptotic protein Bax and caspase-3, and activated the Nrf2/HO-1 signaling pathway to protect ARPE-19 from oxidative stress. In addition, SBP restored mitochondrial morphology and quantity and protected mitochondrial function from oxidative damage by inhibiting mitochondrial permeability and swelling.

## Data Availability

The original contributions presented in the study are included in the article; further inquiries can be directed to the corresponding author.
